# Effect of cholesterol depletion on the pore dilation of TRPV1

**DOI:** 10.1186/1744-8069-9-1

**Published:** 2013-01-02

**Authors:** Erik T Jansson, Carolina L Trkulja, Aikeremu Ahemaiti, Maria Millingen, Gavin DM Jeffries, Kent Jardemark, Owe Orwar

**Affiliations:** 1 Department of Chemical and Biological Engineering, Chalmers University of Technology, SE-412 96 Göteborg, Sweden; 2 Department of Physiology and Pharmacology, Karolinska Institutet, SE-171 77 Stockholm, Sweden

**Keywords:** TRPV1, Cholesterol, MβCD, Capsaicin, Acidic pH, YO-PRO, Ion-permeability, NMDG

## Abstract

The TRPV1 ion channel is expressed in nociceptors, where pharmacological modulation of its function may offer a means of alleviating pain and neurogenic inflammation processes in the human body. The aim of this study was to investigate the effects of cholesterol depletion of the cell on ion-permeability of the TRPV1 ion channel. The ion-permeability properties of TRPV1 were assessed using whole-cell patch-clamp and YO-PRO uptake rate studies on a Chinese hamster ovary (CHO) cell line expressing this ion channel. Prolonged capsaicin-induced activation of TRPV1 with *N*-methyl-D-glucamine (NMDG) as the sole extracellular cation, generated a biphasic current which included an initial outward current followed by an inward current. Similarly, prolonged proton-activation (pH 5.5) of TRPV1 under hypocalcemic conditions also generated a biphasic current including a fast initial current peak followed by a larger second one. Patch-clamp recordings of reversal potentials of TRPV1 revealed an increase of the ion-permeability for NMDG during prolonged activation of this ion channel under hypocalcemic conditions. Our findings show that cholesterol depletion inhibited both the second current, and the increase in ion-permeability of the TRPV1 channel, resulting from sustained agonist-activation with capsaicin and protons (pH 5.5). These results were confirmed with YO-PRO uptake rate studies using laser scanning confocal microscopy, where cholesterol depletion was found to decrease TRPV1 mediated uptake rates of YO-PRO. Hence, these results propose a novel mechanism by which cellular cholesterol depletion modulates the function of TRPV1, which may constitute a novel approach for treatment of neurogenic pain.

## Background

Nociceptors are sensory neurons which respond to noxious stimuli, resulting in the perception of pain. Nociceptors are also involved in neurogenic inflammation, *i.e.* inflammatory symptoms that result from the release of pro-inflammatory substances (*e.g.* the neuropeptide substance P) from these sensory nerve terminals [1,2]. A key mediator of nociception and neurogenic inflammation is the TRPV1 ion channel which is expressed in nociceptors [3-5]. TRPV1 is activated by a broad range of chemical stimuli which includes protons, capsaicin, and arachnoid toxin, but also environmental effects such as painfully hot temperatures (>42°C). TRPV1 mediates an excitatory response to stimuli, *i.e.* it is a nonselective cation channel that is permeable not only for monovalent cations, but also for Ca^2+^ and relatively large cations [3,4,6-9]. TRPV1 has been shown to possess a dynamic selectivity for ions during stimulation similar to what has been observed for P2X purinoceptor channels, and later also for the TRPA1 ion channel, *i.e.* a time- and agonist concentration-dependent increase of the relative permeability of the ion channel to large cations [10-14]. Thus, these physicochemical properties of TRPV1 may contribute to its capability of mediating nociception and neurogenic inflammation.

Neurogenic inflammation is generated through excessive stimulation of the nociceptor terminals within injured or inflamed tissue. As indicated above, the nociceptors are sensitized in this process by the release of pro-inflammatory substances or inflammatory mediators. However, attempts to decrease sensitization of nociceptors, and thereby symptoms of neurogenic inflammation (especially the enhanced pain perception), by using antagonists to pro-inflammatory substances have not been successful, since these mediators are involved in similar actions, or functions [2]. TRPV1 significantly contributes to the integration of these actions of various inflammatory modulators, which makes this ion channel a central regulator of nociceptor excitability. Consequently, the key role of TRPV1 in the onset and maintenance of neurogenic inflammation, has identified it as a potential drug target for alleviating inflammatory pain [15-17].

Pharmacological perturbation of cholesterol synthesis, interfering with ion channel function, may represent a novel therapeutic approach to inhibit TRPV1-mediated responses and sensitization of nociceptors in the process of neurogenic inflammation. Interestingly, recent studies suggest that cholesterol interferes with the function of TRPV1 [18-20], but none of them demonstrate how cholesterol modulation affects the ion-permeability properties of this channel, which we believe may contribute to drastic changes in the sensitization of nociceptors. By using whole-cell patch-clamp recording and laser scanning confocal microscopy (LSCM) studies of uptake rates of the fluorescent dye YO-PRO [10,12], we have here investigated the effects of cellular cholesterol depletion on the dynamic permeability properties of the TRPV1 ion channel. We found that cholesterol depletion impairs the ability of TRPV1 to *N*-methyl-D-glucamine (NMDG) during hypocalcemic conditions, upon sustained activation with capsaicin and to decrease secondary currents upon sustained activation with protons. We also found cholesterol depletion to decrease the cellular uptake rate of YO-PRO via TRPV1, when activated with capsaicin or protons. Thus, our findings suggest that cholesterol depletion modulates the extent of TRPV1 pore dilation under hypocalcemic conditions.

## Results

For electrophysiological experiments and fluorescence studies, a Chinese hamster ovary (CHO) cell line, expressing human TRPV1 channels was used. Methyl-β-cyclodextrin (MβCD) alone, or in combination with cholesterol dissolved in serum-free culturing medium was used to perturbate the cholesterol content of the CHO cells [18,21,22].

### Decrease of cholesterol content in CHO cells

The cholesterol content of CHO cells was quantitated with gas chromatography–mass spectrometry (GC–MS) [23-26], and found to be decreased by both 15 min treatment with 10 mM MβCD or 90 min treatment with 2.5 mM MβCD:cholesterol (10:1). Cells treated with 10 mM MβCD had a cholesterol content of 2.55  ±  0.58 μg/million cells (*n* = 9), MβCD:cholesterol-treated cells had a cholesterol content of 1.82  ±  0.22μg/million cells (*n* = 8), compared to control cells which had a cholesterol content of 4.0  ±  1.4 μg/million cells (*n* = 9), *i.e.* the cholesterol content of the cells was decreased by ∼36% (*p* < 0.05) and ∼54% (*p* < 0.001) using the MβCD or the MβCD:cholesterol treatment, respectively.

### Cholesterol depletion affects NMDG-permeability of TRPV1 in CHO cells

To assess the effect of cholesterol on the pore dilation of TRPV1, voltage ramps were measured during 1 min application of 1 μM capsaicin, in extracellular buffers with either sodium ions, or NMDG as the sole cation (buffer **1** or **2**, respectively), combined with an intracellular buffer mainly containing sodium ions (buffer **3**). NMDG is an amino sugar which becomes cationic at neutral pH.

**Figure 1:**
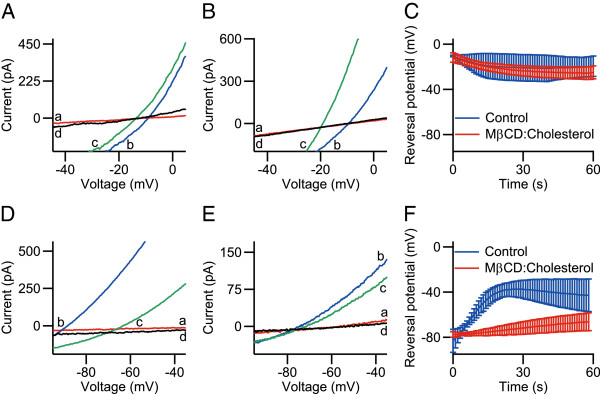
**Effect of cholesterol depletion for 90 min treatment with 2.5 mM MβCD:cholesterol (10:1) on NMDG-permeability of capsaicin-activated TRPV1.** The relative permeability of NMDG with respect to sodium ions in TRPV1 was assessed with whole-cell patch-clamp recording by measuring reversal potentials with voltage ramps ranging from −120mV to + 30mV over 150 ms, upon sustained activation with 1 μM capsaicin. Lower-case letters in panels with representative current-voltage (IV) relations indicate timepoints during the stimulation protocol: (a) before application of capsaicin; (b) first time-point of capsaicin application; (c) late time-point before capsaicin removal; (d) after removal of capsaicin. Panels (A–C) show data from experiments using extracellular sodium ions (extracellular buffer **1**, intracellular buffer **3**). Panels (D–F) show data from experiments using extracellular NMDG (extracellular buffer **2**, intracellular buffer **3**). (**A**) Representative IV-traces of a control cell. (**B**) Representative IV-traces of a cell treated with MβCD:cholesterol. (**C**) Reversal potentials for TRPV1 with extracellular sodium ions during application of capsaicin, no difference of reversal potential was observed between the groups (*n* = 4–6). (**D**) Representative IV-traces of a control cell. (**E**) Representative IV-traces of a cell treated with MβCD:cholesterol. (**F**) Reversal potentials for TRPV1 with extracellular NMDG during application of capsaicin. The reversal potential shift observed in control cells was inhibited by treatment with MβCD:cholesterol (*n* = 4, *p* < 0.05).

No effect on the shift of reversal potential *E*_rev_ was observed for 90 min treatment with 2.5 mM MβCD:cholesterol (10:1) in the extracellular sodium ion-containing buffer (Figure 1A–C). Here, in MβCD:cholesterol-treated cells, *E*_rev_ shifted from −11.7  ±  4.1 mV to −24.9  ±  5.8 mV (*n* = 6), whereas for control cells, *E*_rev_ shifted from −10.35  ±  0.68mV to −19.6  ±  9.0mV (*n* = 4). However, with the extracellular buffer containing NMDG, a significant effect by treatment with MβCD:cholesterol on the final reversal potentials was observed. For MβCD:cholesterol-treated cells, *E*_rev_ shifted from −77.7 ± 2.5 mV to −66.7 ± 7.6 mV (*n* = 4), whereas for control cells, *E*_rev_ shifted from −83 ± 10mV to −43 ± 14mV (*n* = 4, *p* < 0.05, Figure 1D–F). Using eq. 1 (see Methods), the relative permeability *P*_NMDG_ / *P*_Na_ after 20 s of capsaicin application was calculated to be on average 0.12 and 0.46 for MβCD:cholesterol-treated cells and control cells, respectively, *i.e.* a decrease of cellular cholesterol content resulted in a decrease of *P*_NMDG_ / *P*_Na_ in TRPV1 by ∼70%.

### Cholesterol depletion affects the capsaicin-activated current mediated by TRPV1 in CHO cells

**Figure 2:**
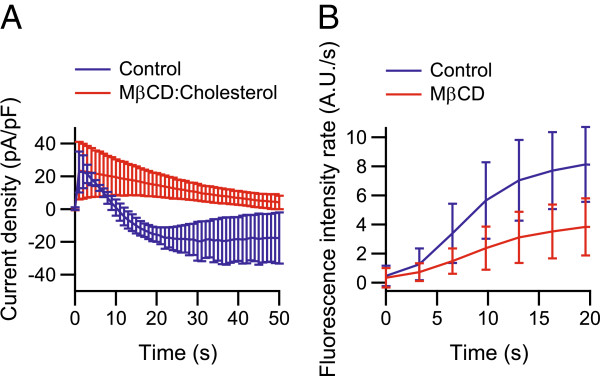
**Effect of cholesterol depletion for 15 min treatment with 10 mM MβCD and for 90 min treatment with 2.5 mM M**β**CD:cholesterol (10:1) on capsaicin-activated TRPV1.** (**A**) Whole-cell patch-clamp recordings for TRPV1 treated with MβCD:cholesterol during application with 1 μM capsaicin from ramp experiments evaluated at a holding potential of −60mV, with extracellular NMDG (buffer **2**), and intracellular sodium ions (buffer **3**). Current densities were initially equal for both the control and treated cells, but the time-dependent switch of ion flow direction observed in control cells was inhibited by treatment with MβCD:cholesterol (*n* = 4, *p* < 0.05). (**B**) YO-PRO uptake was observed during stimulation of MβCD-treated cells with 1 μM capsaicin (extracellular buffer **8**). The fluorescence uptake rate increased during activation with 1 μM capsaicin, and a significant difference in rate was observed, as the uptake rate after ∼20 s of stimulation was reduced by ∼50% in treated cells compared to control cells (*n* = 34–41, *p* < 0.001).

Whole-cell patch-clamp traces for TRPV1 during application of 1 μM capsaicin from voltage ramp experiments were evaluated at a membrane potential of −60 mV, with extracellular NMDG (buffer **2**), and intracellular sodium ions (buffer **3**). Whereas TRPV1 initially mediated an outward current which upon sustained activation changed into an inward current for control cells, the current in cells treated for 90 min with 2.5 mM MβCD:cholesterol (10:1) remained directed outward (Figure 2A). In the MβCD:cholesterol-treated cells, the initial current density induced by capsaicin was 23 ± 18 pA/pF and reduced to 5.2 ± 2.9pA/pF (*n* = 4), remaining outwardly directed, whereas in control cells, the initial current density changed from 22 ± 10 pA/pF to −19 ± 14 pA/pF (*n* = 4). There was no difference between the initial current densities upon activation with agonist, but the late phase current densities were different (*p* < 0.05). Current densities were integrated over the time of application of capsaicin, and showed the net flow of ions to be outward (0.66 ± 0.28 nA·s/pF, *n* = 4) and inward (−0.65 ± 0.50 nA·s/pF, *n* = 4), for MβCD:cholesterol-treated and control cells, respectively (*p* < 0.005).

For cells that were treated for 15 min with 10 mM MβCD, uptake of YO-PRO was observed with LSCM during activation of TRPV1 with 1 μM capsaicin in presence of 1 μM YO-PRO in extracellular buffer **8** (Figure 2B). The fluorescence uptake rate increased during the initial 20 s of stimulation with 1 μM capsaicin in both MβCD-treated cells and control cells. However, after ∼20 s of stimulation the fluorescence uptake rate in MβCD-treated cells was 3.8 ± 2.0 A.U./s (*n* = 41) compared to control cells with a rate of 8.1 ± 2.6A.U./s (*n* = 34). Hence, the MβCD-treatment resulted in a reduction by ∼50% of the uptake rate (*p* < 0.001).

### Cholesterol depletion affects the proton-activated current mediated by TRPV1 in CHO cells

**Figure 3:**
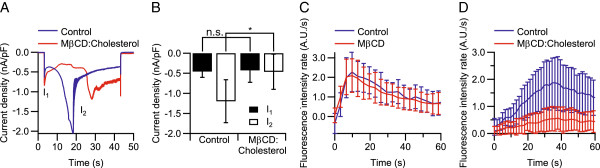
**Effect of cholesterol depletion for 90 min treatment with 2.5 mM M**β**CD:cholesterol (10:1) but not for 15 min treatment with 10 mM MβCD on proton-activated TRPV1.** (**A**) Representative traces of TRPV1 whole-cell response of cells treated with MβCD:cholesterol, activated with pH 5.5, clamped at −60mV (extracellular buffer **4**, intracellular buffer **5**), containing two currents, *I*_1_ and *I*_2_. (**B**) The initial current density upon activation *I*_1_was unaffected by treatment with MβCD:cholesterol whereas the following current density *I*_2_ was 60% lower for cells treated with MβCD:cholesterol compared to control cells (*n* = 6, *p* < 0.05). (**C**) YO-PRO uptake was observed during stimulation with pH 5.5 (extracellular buffer **4**) in cells treated with MβCD. Fluorescence uptake rate increased during activation with pH 5.5, but no significant difference in rate could be determined from the treatment (*n* = 34–46). (**D**) YO-PRO uptake was observed during stimulation with pH 5.5 (extracellular buffer **4**) in cells treated for 90 min with 2.5 mM MβCD:cholesterol (10:1). Fluorescence uptake rate increased during activation with pH 5.5, the MβCD:cholesterol-treatment resulted in a reduction by ∼70% of the uptake rate in treated cells compared to control cells (*n* = 15–30, *p* < 0.001) after ∼30 s of stimulation.

Whole-cell patch-clamp recordings of control cells at −60mV with extracellular buffer **4** (pH 5.5), and intracellular buffer **5** (Figure 3A), showed an initial response followed by desensitization. A second, larger current was also observed which desensitized until it reached a steady-state level. In cells treated for 90 min with 2.5 mM MβCD:cholesterol (10:1), identical initial current responses were observed. However, the magnitude of the second current was smaller. The initial current density *I*_1_ upon activation was −0.42 ± 0.30 nA/pF for treated cells and −0.46 ± 0.15 nA/pF for control cells. Hence, this current was unaffected by treatment with MβCD:cholesterol. The second current density *I*_2_, was −0.46 ± 0.44 nA/pF for treated cells (*n* = 6) and −1.20 ± 0.53 nA/pF for control cells (*n* = 6). Thus, this current density was found to be ∼60% smaller for cells treated with MβCD:cholesterol compared with control cells (*p* < 0.05, Figure 3B).

For cells that were treated for 15 min with 10 mM MβCD, uptake of YO-PRO was observed with LSCM during activation of TRPV1 with pH 5.5 (extracellular buffer **4**) in the presence of 1 μM YO-PRO, using a microfluidic pipette for drug delivery [27,28]. The uptake rate increased during the initial 10 s of stimulation with pH 5.5 where it reached a maximum for both treated and control cells, but no significant difference in uptake rate compared to control cells was observed. The uptake rate was 2.09 ± 0.86 A.U./s (*n* = 46) and 2.3 ± 1.0 A.U./s (*n* = 34) for treated and control cells, respectively (Figure 3C).

In cells treated for 90 min with 2.5 mM MβCD:cholesterol (10:1), a change in uptake rate of YO-PRO could also be observed during activation of TRPV1 with pH 5.5 (extracellular buffer **4**) in the presence of 1 μM YO-PRO, using the microfluidic device for rapid well perfusion. However, here the uptake rate was ∼70% less than the rate observed in control cells (Figure 3D), as the uptake rate after ∼30 s of activation with pH 5.5 was 0.55 ± 0.45 A.U./s (*n* = 30) in cells treated with MβCD:cholesterol compared to an uptake rate of 1.87 ± 0.91 A.U./s (*n* = 15) in control cells (*p* < 0.001).

### No effect of cholesterol depletion on heat-activated current mediated by TRPV1 in CHO cells

**Figure 4:**
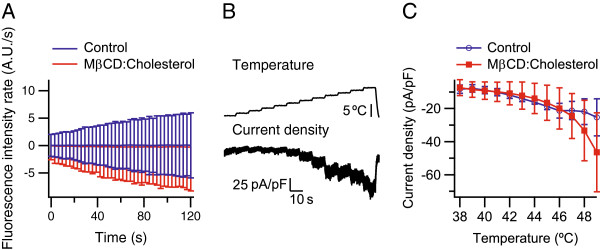
**No effect of cholesterol depletion for 90 min treatment with 2.5 mM MβCD:cholesterol (10:1) on heat-activated TRPV1 currents.** (**A**) The uptake rate of YO-PRO mediated by TRPV1 during sustained activation with heat (45°C) of cells in extracellular buffer **8**, was unaffected by treatment with MβCD:cholesterol (*n* = 80–94). (**B**) Representative trace of a TRPV1 whole-cell response (bottom) from a cell clamped at −60mV (extracellular buffer **6**, intracellular buffer **7**), elicited by a heat-protocol (top) where temperature was ramped from room temperature (∼20°C) to 38°C, then stepped upwards by 1°C to 49°C, held at 10 s per step. (**C**) Heat-activated TRPV1 current densities were unaffected by treatment with MβCD:cholesterol (*n* = 5–9).

Uptake of YO-PRO was measured with LSCM during sustained activation of TRPV1 with heat (45°C), in cells under hypocalcemic conditions (extracellular buffer **8**) and in presence of 1 μM YO-PRO. No increase of the uptake rate of YO-PRO during heat-activation was observed in control cells (*n* = 94). Also, there was no difference of uptake rates between cells treated for 90 min with 2.5 mM MβCD:cholesterol (10:1) (*n* = 80) and control cells (Figure 4A). Further, whole-cell patch-clamp recordings of TRPV1-mediated currents in CHO cells generated by heat, where temperature was ramped from room temperature (∼20°C) to 38°C, then stepped upwards by 1°C to 49°C, held at 10 s per step, at −60 mV in the presence of extra- and intracellular calcium (buffers **6** and **7**, respectively), showed that these current responses were unaffected by treatment of the cells for 90 min with 2.5 mM MβCD:cholesterol (10:1). At each temperature, the amplitudes of the steady-state current densities in cells treated with MβCD:cholesterol (*n* = 9) were similar to those of control cells (*n* = 5, Figure 4B–C).

## Discussion

In accordance with a previous study by Caterina and co-workers [12], our data demonstrates that, under hypocalcemic conditions, prolonged agonist-induced activation of the TRPV1 channel leads to a time-dependent increase of permeability of the channel to large cations. It has been proposed that conformational changes, and not only a rigid pore enlargement, may contribute to the dynamic ionic selectivity of the channel [12]. The major finding of our study is that a decrease of the cholesterol content by ∼54% in the cell inhibits the time-dependent increase of the permeability of the large cation NMDG in the TRPV1 channel under hypocalcemic conditions.

Treatment of cells with 10 mM MβCD and 2.5 mM MβCD:cholesterol (10:1) was quantitated with GC–MS, showing a decrease in cholesterol content for the treated cells by ∼36% and ∼54%, respectively. For cells treated with MβCD:cholesterol, the reversal potential changed with extracellular NMDG during activation of TRPV1 with 1 μM capsaicin, whereas the reversal potential for extracellular sodium ions was unaffected (Figure 1). The relative permeability of NMDG with respect to sodium ions was decreased by ∼70%, due to reduction of the amount of cholesterol in the cell. In measurements with extracellular NMDG the currents were initially directed outward upon activation with 1 μM capsaicin, which can be explained by NMDG being too large to pass through the ion channel. Upon sustained activation with 1 μM capsaicin under hypocalcemic conditions, the pore of TRPV1 expands, eventually allowing for entry of NMDG, resulting in an inward current. This expansion of the pore was inhibited through treatment with MβCD:cholesterol, as the change-of-sign of TRPV1-mediated current observed in control cells was made absent by cholesterol depletion (Figure 2A). The net flow of ions through TRPV1 into the cell during the time of application of capsaicin was found to be outward and inward for MβCD:cholesterol-treated and control cells, respectively. When TRPV1 was activated with pH 5.5 in cells treated with MβCD:cholesterol, the larger second current densities, arising during sustained activation, were found to be decreased by ∼60% (Figure 3A–B).

As an independent assay for probing TRPV1 pore dilation, YO-PRO uptake during stimulation with 1 μM capsaicin or pH 5.5 was measured with LSCM [12,27] on cells treated with 10 mM MβCD or 2.5 mM MβCD:cholesterol (10:1). Whereas a milder cholesterol depletion by ∼36%, using 10 mM MβCD, was unable to affect the uptake rate of YO-PRO when activating TRPV1 with pH 5.5 (Figure 3C), it was found to decrease the uptake rate by ∼50% when TRPV1 was activated with 1 μM capsaicin (Figure 2B). However, a cholesterol depletion of ∼54%, using 2.5 mM MβCD:cholesterol (10:1), was shown to decrease the uptake rate by ∼70% when TRPV1 was activated with pH 5.5 (Figure 3D). This data suggests that there exists a required level of cholesterol depletion for the inhibition of TRPV1 pore dilation, which varies with type of agonist. Also, these results further support the effect of cholesterol depletion on TRPV1 permeability properties observed with patch-clamp recordings, during sustained application of agonist under hypocalcemic conditions.

Our results differ compared to a previous study which demonstrates that cholesterol depletion with a removal of 95% of endogenous cholesterol results in a decreased amount of the TRPV1 protein in the cell membrane, suggesting that this protein might be localized in cholesterol-rich microdomains or lipid rafts in nociceptors [18]. In our experiments, the cholesterol content was decreased to a lesser extent, which seems to change the permeability properties of TRPV1, but not the amount of TRPV1 protein in the cell membrane. If activated with heat, or in the presence of extracellular calcium, permeability changes are not induced upon sustained activation in TRPV1 [12]. Here, as a control, depletion of cholesterol had no effect on the uptake rate of YO-PRO under hypocalcemic conditions, or on the current response in the presence of calcium, mediated by heat-activated TRPV1 (Figure 4). This is in agreement with a previous study where cholesterol depletion was shown to neither have any effect on activation, nor on sensitivity, of heat-elicited TRPV1 currents [22]. This is also supported by our finding that the initial current densities were equal between cells treated with MβCD:cholesterol and control cells in measurements of TRPV1 with 1 μM capsaicin as agonist and NMDG as the sole extracellular cation (Figure 2A). Moreover, whole-cell patch-clamp measurements, using protons (pH 5.5) for activation of TRPV1, indicated that the amount of functional ion channels in the cell membrane was unaffected by the MβCD:cholesterol treatment. Here, the initial current densities remained unchanged, and only the second current densities (arising from pore dilation) were inhibited by treatment upon activation with pH 5.5 (Figure 3A–B). Hence, as initial current densities were similar for both activation with pH 5.5 and 1 μM capsaicin, together with the observation of similar heat-elicited uptake rates of YO-PRO and current densities in treated and control cells, we suggest that the cholesterol depletion did not affect the number of functional channels in the plasma membrane. Furthermore, we also suggest that an effect of cholesterol depletion under hypocalcemic conditions is only observed in TRPV1 current responses associated with an ionic selectivity change of TRPV1.

Another previous study showed that modulation of cholesterol content inhibited TRPV1 function through binding to specific sites along the S5 helix of the ion channel which include a cholesterol recognition/interaction amino acid consensus [20]. However, none of the mentioned studies connect modulation of cellular cholesterol content with the dynamic ionic selectivity which can be induced during prolonged exposure of TRPV1 to an agonist. Thus, our findings suggest that cellular cholesterol depletion interferes with the time-dependent increase of the relative cation permeability of TRPV1 [12], found during sustained activation with capsaicin or protons. This may inhibit the ability of TRPV1 channels to mediate changes of potentials across the membranes of nociceptors.

## Conclusions

We have demonstrated a novel mechanism whereby a decrease of the cholesterol content of a cell affects the ionic selectivity of TRPV1. Prolonged agonist-mediated activation of TRPV1 channels in cells under hypocalcemic conditions may constitute a mechanism which facilitates nociception and neurogenic inflammation, and our results indicate that cholesterol depletion can inhibit these processes. Our data further supports previous investigations, suggesting that cholesterol depletion of the cell modulates the TRPV1 ion channel, which, in turn, may ameliorate pain and inflammation.

## Methods

### Cell culture

Adherent Chinese hamster ovary (CHO) cells with a tetracycline regulated expression system (T-REx) for inducible expression of human TRPV1 were a kind gift from Astra Zeneca, R&D, CNS & Pain, Södertälje, Sweden, and were cultured in T25-flasks and Petri dishes for 2–6 days in medium (DMEM or DMEM/Ham’s F12 with glutamine) supplemented with 10% fetal calf serum, zeocin (350 μg/ml) and blasticidin (5 μg/ml). 18–24 hours prior to experiments, the cells were incubated in medium supplemented with 10% fetal calf serum and doxycycline (1 μg/ml) in order to induce expression of the TRPV1 protein.

### Cholesterol depletion treatment

The 2.5 mM MβCD:cholesterol (10:1) solution was prepared with the direct addition of cholesterol and MβCD in the appropriate molar ratio, into serum-free medium. The solution was incubated over night at 37°C on a rotary shaker, and was filtered through a 0.45 μm filter before use. The 10 mM MβCD solution was prepared by immediate dissolution of MβCD into an appropriate volume of serum-free medium. Prior to experiments, cells were washed with serum-free solution followed by an incubation period with either 15 min of 10 mM MβCD or 90 min of 2.5 mM MβCD:cholesterol (10:1).

### Gas-chromatographic analysis

Cells treated with either 10 mM MβCD or 2.5 mM MβCD:cholesterol (10:1), and control cells only cultured in medium, were detached with Accutase and pelleted. The supernatant was discarded, the cell pellet was thereafter washed with phosphate-buffered saline (PBS) and centrifuged (5 min, 600×g) twice. Before the final centrifugation step, an aliquot was collected for cell counting, which was performed with a Bürker hemocytometer. Cholesterol was extracted from cell pellets by incubation with a hexane/isopropanol (3:2) solution for 4 h. A solution of 5*α*-cholestane in chloroform (50 μl, 0.5 mg/ml) was added to all samples as internal standard, thereafter samples were dried with nitrogen. Before gas-chromatographic measurements, the dry samples were dissolved in 50 μl Sylon HTS and incubated for 30 min at 65°C, whereafter 50 μl of chloroform was added. Cholesterol content of cells without saponification was determined using an HP 5950/5972 gas-chromatography mass-spectrometer (GC–MS) [23-26]. One microlitre (1 μl) of the analyte was injected for analysis, and was separated on an RTX-20 column (Restek, U.S.A.). The inlet temperature was set to 250°C, helium was used as carrier gas, the oven was on hold for 5 min at 250°C, then ramped 5°C/min to 260°C were it remained on hold, and the detector was hold at 300°C. Total ion mass spectra were recorded in a mass over charge (*m*/*z*) range of 50–550, total time for GC–MS detection was 20 min.

### Chemicals

Cell culturing medium (DMEM and DMEM/Ham’s F12 with glutamine), fetal bovine serum, and Accutase were purchased from PAA, Pasching, Austria; Zeocin and YO-PRO-1 were purchased from Invitrogen. All other chemicals were purchased from Sigma. Buffers contained (in mM): **1**, 150 NaOH, 5 EGTA, 10 HEPES; pH adjusted to 7.4 with HCl. **2**, 150 NMDG, 10 EGTA, 10 HEPES; pH adjusted to 7.4 with HCl. **3**, 150 NaOH, 1 MgCl_2_, 5 EGTA, 10 HEPES; pH adjusted to 7.4 with HCl. **4**, 140 NaCl, 5 KCl, 1 MgCl_2_, 10 HEPES, 10 D-glucose, 10 Na_4_BAPTA; pH adjusted to 5.5 with HCl. **5**, 120 KCl, 2 MgCl_2_, 10 Na_4_BAPTA, and 10 HEPES; pH adjusted to 7.2 with KOH. **6**, 140 NaCl, 5 KCl, 1 CaCl_2_, 1 MgCl_2_, 10 Hepes, 10 D-glucose; pH adjusted to 7.4 with NaOH. **7**, 120 KCl, 1 CaCl_2_, 2 MgCl_2_, 11 EGTA, and 10 HEPES; pH adjusted to 7.2 with KOH. **8**, 140 NaCl, 5 KCl, 1 MgCl_2_, 10 HEPES, 10 D-glucose, 10 Na_4_BAPTA; pH adjusted to 7.4 with NaOH. PBS, 136 NaCl, 2.7 KCl, 10 NA_2_HPO_4_, 1.8 KH_2_PO_4_; pH adjusted to 7.4 with NaOH.

### Electrophysiology

All electrophysiological experiments were performed using a microfluidic device for patch-clamp (Dynaflow, Cellectricon AB, Göteborg, Sweden). Data was recorded using a HEKA EPC10 patch-clamp amplifier with Patchmaster software (HEKA Elektronik, Germany). Pipettes were pulled from borosilicate capillaries (GC-150F, Harvard Apparatus, U.K.) with a resistance of 1–12 M*Ω*. Cells were clamped at −60 mV and series resistance compensation was performed up to 80%. Voltage ramp protocols were executed with a rate of 1 Hz, with −60 mV as resting potential and ranged from −120mV to +30 mV over 150 ms. Whole-cell patch-clamp recordings were sampled at 5 kHz and low pass filtered at 1 kHz. Data was analyzed with Fitmaster (HEKA Elektronik, Germany) and Matlab (Mathworks, MA, U.S.A.).

The relative permeability *P*_NMDG_ / *P*_Na_can be derived from the Goldman–Hodgkin–Katz equation, and is calculated as

(1) $$\frac{P_{\text{NMDG}}}{P_{\text{Na}}}=\frac{{\left[{\text{NMDG}}^{+}\right]}_{o}}{{\left[{\text{Na}}^{+}\right]}_{o}}\text{exp}\frac{\Delta E_{\text{rev}}F}{\text{RT}}$$

where *F* is the Faraday constant, *R* is the gas constant, Δ*E*_rev_ is the difference in *E*_rev_ between extracellular NMDG^+^ and Na^+^ measured in parallel cells, *T* is the temperature, and [NMDG^+^]_o_ and [Na^+^]_o_ are the activities of extracellular NMDG^+^ and Na^+^, respectively.

### Temperature control

For electrophysiological experiments, resistive heating coils (2 nm of Cr followed by a 10 nm transparent Au film) were evaporated onto the glass underside of the Dynaflow chip, as previously described [29,30]. Heating was achieved with a current limiting standard power supply with an accuracy of  ± 0.2°C. Cooling was achieved by simple heat dissipation after turning off the heating stage. The temperature in the flow close to the patch-clamped cell was measured using a calibrated micron-sized Cu/Ni thermoelement (Omega Ltd., U.K.).

For fluorescence measurements, heating of cells was achieved with a 5 W 1470 nm (IR-B) semiconductor diode laser (Seminex HHF-1470-6-95, Seminex, Peabody, MA, U.S.A.) used in conjunction with a 50 μm core diameter, 0.22NA optical fiber (Ocean Optics, Dunedin, FL, U.S.A.), and the temperature close to the cells was determined using a thermoelement, as described above.

### YO-PRO uptake assay

YO-PRO uptake was measured with fluorescence using a Leica IRE2 confocal microscope equipped with a Leica TCS SP confocal scanner, an oil-immersion 40× NA 1.3 Leica HCX PL APO CS objective, and Leica confocal software (Leica Microsystems GmbH, Germany). Fluorescence was measured from single cells, with excitation at 488 nm and emission at either 505600 nm or 555–625 nm, images collected at a rate of 0.3–0.6 Hz. Stimulation of cells with agonists together with 1 μM YO-PRO was performed using either a microfluidic pipette (Avalance Biotech AB, Göteborg, Sweden), providing perfusion within a confined volume in the cell dish [27,28], or with a microfluidic device providing rapid well perfusion (Auto-GP, Cellectricon AB, Göteborg, Sweden). Briefly before the onset of agonist, cells were perfused with YO-PRO in the absence of agonist to assure the cell membranes to be intact. Data was analyzed with Leica confocal software and Matlab (Mathworks, MA, U.S.A.).

### Statistical analysis

All statistical analysis was performed with Student’s paired or unpaired *t*-test where applicable. Results were considered statistically significant if *p* < 0.05, and indicated with an asterisk (*) in Figures where applicable. Data is presented as mean plus/minus one standard deviation.

## Competing interests

The authors declare that they have no competing interests.

## Authors’ contributions

ETJ planned and participated in conducting the experiments, performed data analysis and wrote the manuscript. CLT, AA, MM and GDMJ participated in conducting experiments and data analysis. KJ and OO supported the study and participated in writing the manuscript. All authors read and approved the final manuscript.
